# Hypermanganesemia due to mutations in *SLC39A14*: further insights into Mn deposition in the central nervous system

**DOI:** 10.1186/s13023-018-0758-x

**Published:** 2018-01-30

**Authors:** L. Marti-Sanchez, J. D. Ortigoza-Escobar, A. Darling, M. Villaronga, H. Baide, M. Molero-Luis, M. Batllori, M. I. Vanegas, J. Muchart, L. Aquino, R. Artuch, A. Macaya, M. A. Kurian, Pérez Dueñas

**Affiliations:** 1 Department of Biochemistry, Institut de Recerca - Hospital Sant Joan de Déu, University of Barcelona, Barcelona, Spain; 2 Department of Child Neurology, Institut de Recerca - Hospital Sant Joan de Déu, University of Barcelona, Barcelona, Spain; 3 Department of Pharmacy, Institut de Recerca - Hospital Sant Joan de Déu, University of Barcelona, Barcelona, Spain; 4 Department of Radiology, Institut de Recerca - Hospital Sant Joan de Déu, University of Barcelona, Barcelona, Spain; 5 Deparment of Pediatrics, Hospital de Mataró, Barcelona, Spain; 6 Pediatric Neurology Research Group, Vall d’Hebron Research Institute (VHIR), Universitat Autònoma de Barcelona, Passeig de la Vall d’Hebron, 119-129, 08035 Barcelona, Catalonia Spain; 7 Molecular Neurosciences, Developmental Neurosciences Programme, UCL-Great Ormond Street Institute of Child Health, London, UK

**Keywords:** Manganese homeostasis, *SLC39A14*; *SLC30A10*, Dystonia, Pallidum, Hypermanganesemia

## Abstract

**Background:**

The *SLC39A14*, *SLC30A10* and *SLC39A8* are considered to be key genes involved in manganese (Mn) homeostasis in humans. Mn levels in plasma and urine are useful tools for early recognition of these disorders. We aimed to explore further biomarkers of Mn deposition in the central nervous system in two siblings presenting with acute dystonia and hypermanganesemia due to mutations in *SLC39A14*. These biomarkers may help clinicians to establish faster and accurate diagnosis and to monitor disease progression after chelation therapy is administered.

**Results:**

A customized gene panel for movement disorders revealed a novel missense variant (c.311G > T; p.Ser104Ile) in *SLC39A14* gene in two siblings presenting at the age of 10 months with acute dystonia and motor regression. Mn concentrations were analyzed using inductively coupled mass spectrometry in plasma and cerebrospinal fluid, disclosing elevated Mn levels in the index case compared to control patients. Surprisingly, Mn values were 3-fold higher in CSF than in plasma. We quantified the pallidal index, defined as the ratio between the signal intensity in the globus pallidus and the subcortical frontal white matter in axial T1-weighted MRI, and found significantly higher values in the *SLC39A14* patient than in controls. These values increased over a period of 10 years, suggesting the relentless pallidal accumulation of Mn. Following genetic confirmation, a trial with the Mn chelator Na_2_CaEDTA led to a reduction in plasma Mn, zinc and selenium levels. However, parents reported worsening of cervical dystonia, irritability and sleep difficulties and chelation therapy was discontinued.

**Conclusions:**

Our study expands the very few descriptions of patients with *SLC39A14* mutations. We report for the first time the elevation of Mn in CSF of *SLC39A14* mutated patients, supporting the hypothesis that brain is an important organ of Mn deposition in SLC39A14-related disease. The pallidal index is an indirect and non-invasive method that can be used to rate disease progression on follow-up MRIs. Finally, we propose that patients with inherited defects of manganese transport should be initially treated with low doses of Na_2_CaEDTA followed by gradual dose escalation, together with a close monitoring of blood trace elements in order to avoid side effects.

## Background

Manganese (Mn) is a trace metal with a key role as a cofactor of multiple enzymes, including hydrolases, lyases, glycosyltransferases, arginase, glutamine synthase and superoxide dismutase (SOD), in the synthesis of hormones and neurotransmitters [1] and during inflammatory events of the central nervous system [2].

Mn deposition in the brain can occur due to acquired causes (such as environmental exposure), as well as inherited defects in Mn transport and metabolism. Mn intoxication has been described in miners, welders, individuals working or living near ferro-alloy factories or in those drinking contaminated water, as well as in patients receiving total parenteral nutrition or those with acquired hepatocerebral degeneration [3]. Patients with severe Mn exposure may develop an extrapyramidal syndrome termed manganism, with rigidity, bradykinesia and dystonia [3]. Mn dyshomoeostasis may also result from inherited genetic defects in one of the transporters implicated in Mn homeostasis, namely *SLC39A8*, *SLC30A10* and *SLC39A14*. Recently, several in vitro and in vivo models have elucidated their role in the transport of Mn and other divalent metals [2, 4–10]*. SLC39A8* (MIM608732) encodes ZIP8, a Mn and Zn transporter that localizes to the hepatocyte canalicular membrane and reclaims Mn from bile [7]. Knock out ZIP8 mice showed markedly decreased Mn levels in multiple organs and whole blood, increased bile levels, and decreased activity of Mn-dependent enzymes, such as arginase and β-1, 4-galactosyltransferase [7]. Biallelic mutations in this gene lead to severe Mn depletion and a secondary congenital disorder of glycosylation (CDG) syndrome. Patients manifest developmental delay and intellectual disability, dwarfism, craniosynostosis, cerebellar atrophy, seizures and Leigh-like syndrome [11, 12].

*SLC30A10* (also known as ZnT10) (MIM611146) and *SLC39A14* (also known as ZIP14) (MIM608736) are efflux and influx transporters, respectively, that cooperatively regulate Mn homeostasis in humans. Recently, Liu et al. 2017 generated knockout (KO) mice models lacking *SLC30A10*, *SLC39A14* and both transporters (double knockouts) demonstrating high blood and brain Mn levels in the three mice models, but only high liver Mn levels in the single *SLC30A10* KO model. These findings are in agreement with those observed in patients with recessive mutations in *SLC30A10* and *SLC39A14*, showing cerebral Mn deposition as a consequence of increased systemic Mn load in both disorders [13], but only polycythemia and liver cirrhosis in *SLC30A10* [14].

Animal models demonstrated that SLC30A10 and SLC39A14 localized to the canalicular and basolateral domains of hepatocytes, respectively, thereby mediating Mn biliary excretion synergistically [8]. Moreover, *SLC39A14* KO mice showed reduce Mn transport into enterocytes at the basolateral membrane, thereby decreasing Mn excretion via the gastrointestinal tract [4].

Patients with *SLC30A10* and *SLC39A14* defects show a progressive dystonia-parkinsonism syndrome as a consequence of Mn toxicity to the basal ganglia. Chelation therapy in both disorders increases Mn urinary excretion and decreases plasma Mn concentrations with variable clinical improvement [14–17]. *SLC39A14* KO mice suffer from Mn brain deposition and motor dysfunction, thus recapitulating the disease in humans [4, 10].

In this study, we present two siblings with homozygous *SLC39A14* mutations causing hypermanganesemia, progressive dystonia, severe disability and early death. Our study reports a likely novel pathogenic variant in *SLC39A14*, thereby expanding the reported mutations in this gene. We examine the levels of Mn and other trace elements in plasma and cerebrospinal fluid (CSF), as potential biomarkers for diagnosis and treatment monitoring. We also describe serial magnetic resonance imaging (MRI) abnormalities over time, confirming progressive changes with pallidal Mn deposition.

## Methods

The index case was born to healthy consanguineous Senegalese parents. Detailed delineation of the patient’s history, disease course and clinical examination was undertaken, as well as molecular genetics, radiological and biochemical studies. Family history revealed a similarly affected older brother for whom a stored DNA sample was available. Parental DNA was also obtained. The study was approved by the ethics committee at Sant Joan de Déu Hospital and parents gave written informed consent for study participation.

We also performed a literature review on genetic causes of Mn dysregulation, by searching MEDLINE (through PubMed) the following keywords: #1 *SLC30A10*, #2 *SLC39A14*, #3 *SLC39A8*, #4 hypermanganesemia and #5 manganese homeostasis. A total of 12 clinical studies were finally selected (Table 1) [11, 12, 15–25].

**Table 1:** Characteristics of patients with *SLC30A10*, *SLC39A14*, and *SLC39A8* mutations

Phenotypes	*SLC30A10*	*SLC39A14*	*SLC39A8*
	Early-onset dystonia, polycythemia and hepatopathy, adult-onset parkinsonism and spastic paraparesis	Rapidly progressive childhood-onset parkinsonism-dystonia	Type II congenital disorder of glycosylation with Leigh syndrome and autosomal recessive intellectual disability with cerebellar atrophy
Number of patients reported	39	10	12
References	[16–24]	[15] current paper	[11, 12, 25]
First described in	2000	2016	2015
Age at onset, median (IQR)	7.1 (1–57 years)	15.8 (7–36 months)	Birth to 1 year of age
Sex	20F/19M	6F/4M	8F/4M
Death and cause	4 death (3 cirrhosis-related complications and 1 pneumonia)	4 death (2 respiratory infections and 2 unknown cause)	1 death (infection)
Parental Consanguinity (N)	34	10	10
Main neurological signs and symptoms	Focal and generalized dystonia, gait disturbances “cock-walk gait” and Parkinsonism	Generalized dystonia and Parkinsonism	Profound hypotonia
Other neurological signs and symptoms	Central hypotonia, behavioral changes, developmental delay, dysphagia, ataxia, spastic paraparesis and sensory motor axonal polyneuropathy	Spasticity, developmental delay, bulbar dysfunction	Dystonia, opisthotonus, severe intellectual disability, strabismus, nystagmus, hearing impairment, apnea/hypopnea episodes, axonal neuropathy, generalized and myoclonic seizures and infantile spasm
Abnormal head growth / skull deformity	Normal head circumference	Microcephaly (*N* = 4), macrocephaly (*N* = 1), Craniosynostosis (N = 1)	Normal head circumference, craniosynostosis in 1 patient
Blood Mn levels (nmol/L)	Increased 3345.7 ± 2575.3 (RV: <320)	Increased 2898 ± 2532(RV: <320)	Decreased 16.4 ± 5.8 (RV: 5.3–40.8)
Urinary Mn levels	Increased 11.3 ± 4.8 mcg/L (RV: 0.5–4)	Not reported (increased in our patient: 8.2 mcg/L; RV:0.4–0.9)	Increased 56.5 ± 73.2 nmol/L (RV: 1.3–9.1)
Systemic involvement and others biochemical abnormalities	Hepatopathy: Hepatomegaly in 14 patients, liver cirrhosis in 8 patients and increased transaminases in 41%: ALT: 107.1 ± 50.7 (RV <55) Polycythemia in 21% of patients: haematocrit 52.8 ± 6.4% (RV: 34–40)	Not reported	Dysmorphic features^a^, dwarfism with short limbs and scoliosis Increased transaminases in 2 patient (AST: 441 UI/L (RV < 80), ALT: 102 and 113 UI/L (RV < 55)) and impaired blood coagulation 1 patient High blood lactate (8.7 mmol/L) and CSF lactate (4.2 mmol/L) in 1 patient (RV: <1.9) Abnormal glycosylation pattern in 7 patients
Brain MRI	T1 W hyperintensity Basal ganglia 38 Thalamus 20 Brainstem 13 Cerebellum 21 Pituitary gland 6	T1 W hyperintensity Basal ganglia 10 Pituitary gland 8 Cerebral white matter 10	T2 W hyperintensity Basal ganglia 2
	Brainstem atrophy 1	Diffuse cerebral and cerebellar atrophy 4	Diffuse cerebellar atrophy 10 Frontal lobes atrophy 1
Genetics findings	Missense 5 Stop gained 3 Deletion 11 Splicing 1	Missense 8 Stop gain 1 Deletion 1	Missense 14
	Homozygous 37 Heterozygous 0	Homozygous 10 Heterozygous 0	Homozygous 10 Heterozygous 2
Chelation therapy	Disodium calcium edetate, calcium ethylenediaminetetra-acetic acid, D-penicilamina and 2,3 mercaptosuccinic acid	Disodium calcium edetate	
Other Treatments	Iron oral supplementation 19 Zinc, vitamins C and D supplementation, manganese free-diet, L-dopa, pramiprexole and intratechal baclofen		Galactose, manganese, CoQ10, thiamine, pyridoxine and glucocorticoid

*F* Female, *M* Male, *Mn* Manganese, *IQR* Interquartile range, *RV* Reference values

^a^Dysmorphic features include a broad forehead, mid-face hypoplasia, small jaw, hirsutism, anteverted nostrils, thin lips and a smooth philtrum

### Biochemical studies

CSF and plasma samples were analyzed with an Agilent 7500ce inductively coupled plasma mass spectrometer (Agilent Technologies, Waldbronn, Germany). The instrument uses a collision/reaction cell with hydrogen for selenium (Se) determination, and helium for zinc (Zn) and Mn. Briefly, after ionization of plasma or CSF samples in the plasma torch and elimination of interference in the collision/reaction cells, element concentrations were determined by mass spectrometry, as previously reported [26, 27]. Hemolysed plasma samples were excluded to avoid blood contamination that can significantly increase Zn and Mn values.

*Plasma samples preparation*: 25 μL of plasma samples were diluted (1:40; V:V) in 25 μL of distilled water and 950 μL of a solution containing 0.7 mmol/L EDTA (Merck, Darmstadt, Germany), 0.07% Triton-X-100 (Merck), 2% butanol, 0.5% ammoniac (Merck) and germanium as internal standard (Merck).

*CSF samples preparation*: 50 μL of CSF samples were diluted (1:20; V: V) in a 2% nitric solution and 10 μg/L of germanium as internal standard (Merck).

### MRI studies

The pallidal index (PI), defined as the ratio between the signal intensity in the globus pallidus (SIGP) and the subcortical frontal white matter (SIFW) in axial T1-weighted MRI [28] was calculated in our patient on MRIs undertaken at age 11 months, 8 and 10 years. Values were compared to nine age-matched control patients. The MRIs in control patients were obtained as part of a diagnostic protocol for patients with chronic headache, and were classified as normal by expert neuroradiologists.

### Genetic analysis

A customized gene panel for movement disorders was design by Sure Design Tool (Agilent Technologies, Santa Clara, CA, USA). This panel included 78 genes causing basal ganglia disease which were classified in four groups: Aicardi-Goutières syndrome, thiamine metabolism, mitochondrial disorders and other neurometabolic disorders, including *SLC30A10* and *SLC39A14* related to Mn dysregulation. Library construction was performed according to manufacturer’s protocol using HaloPlex technology. Sequencing was carried out on MiSeq sequencer (Illumina, San Diego, CA, USA). Data processing, variant calling and variant annotation were done by DNAnexus platform and Variant Studio software. The average of mean-coverage in the sample gene panel was 95% for a read depth of 20X. Filtering was performed by minor allele frequency < 1% and possible pathogenicity based on mutation effects (frameshift, insertions deletions, missense, stop gain and splice site regions). Variant validation and segregation studies were done by PCR with Sanger sequencing using the Big Dye Terminator Cycle Sequencing System (Applied Biosystems). Primers for validation of the identified change in *SLC39A14* were forward primer 5’-GAAGGCTGAGTAGGTTGCTG- 3′ and reverse primer 5’-CTCCTCGTTTTCCTGGTTCT-3′.

## Results

### Clinical presentation

The proband was born from consanguineous healthy Senegalese parents, after an uneventful pregnancy and delivery. He had a normal perinatal period and early neurodevelopmental milestones were on average. At 11 months he developed acute generalized dystonia and neurological regression following an intercurrent viral respiratory infection. On neurological examination there was evidence of skull deformity with normal head circumference, dystonic tetraparesis, oromandibular dystonia and opisthotonos. A plain skull radiogram revealed multiple-suture craniosynostosis. Metabolic investigations in blood, CSF and urine were normal at that moment, except for a mild decrease in 5-hydroxyindolacetic acid concentrations (104 nmol/L, reference values (RV): 170–490). The family moved to Senegal and they returned to our hospital at the age of 9 years. At this time, the patient had developed microcephaly (head circumference 51 cm, 6th percentile), severe dystonic tetraparesis, anarthria, dysphagia and malnutrition. He required enteral tube feeding and received baclofen, diazepam and gabapentin for symptomatic control of dystonia.

On reviewing the family history, it became apparent that there was a similarly affected older brother, born in 1997, who developed acute dystonic tetraparesis associated with rigidity, hypokinesia and pyramidal signs, following a viral illness at the age of 10 months. Brain MRI revealed bilateral pallidal T2-hypointensity and pallidal and cerebral and cerebellar white matter T1-hyperintensity. His clinical status remained unchanged until age 21 months, when he died in Senegal of unknown cause.

The clinical picture of these siblings was analyzed in the context of the existing literature on 46 patients previously identified with genetic defects leading to Mn dysregulation (Table 1).

### Biochemical studies

Biochemical studies in the proband detected normal full blood count, liver function, Fe metabolism, Zn, Se and copper (Cu) concentrations in plasma. We also found elevated plasma Mn (10.5 μg/L, RV: 0.4–0.9 μg/L) and extremely elevated CSF Mn concentrations (34 μg/L, RV: 0.5–1.7 μg/L).

### MRI analysis

**Fig. 1:**
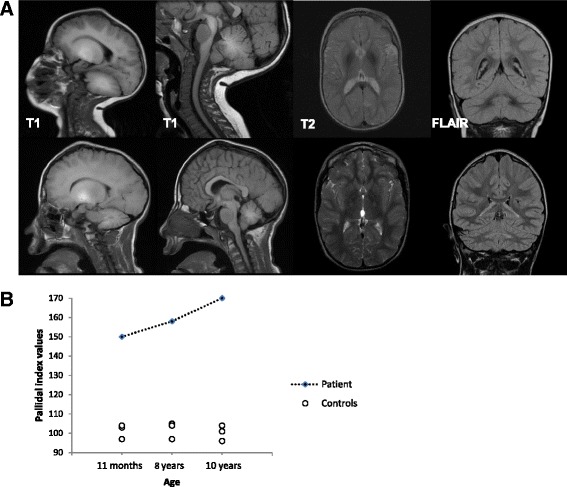
Radiological findings from the patient. **a**: Brain MRI of the patient at 11 months (first line) shows a high-T1 and low T2 signal in the pallidi and dentate nuclei. MRI at 10 years (second line) shows persistent T1-hyperintensity of the globi pallidi, volume loss, gliosis and atrophy of the dentate nuclei and moderate atrophy of cerebellar folia. **b**: Distribution of individual PI scores in patient and controls

Brain MRI of the patient at 11 months showed a symmetrical high-T1 and low T2 signal in both the pallidum and dentate nuclei. White matter signal intensity of the cerebrum, cerebellum and brainstem was also very high in T1 (Fig. 1a). Follow-up MRI at 10 years showed persistent T1-hyperintensity of the globi pallidi, volume loss, gliosis and atrophy of the dentate nuclei and moderate atrophy of cerebellar folia. Moreover, quantitative assessments of the PI was significantly higher in the patient at age 11 months, 8 and 10 years as compared to nine age-matched controls (U = 0.000; *p* = 0.009, Mann-Whitney U test). We also observed slightly increased signal intensity over time (Fig. 1b).

### Genetic analysis

**Fig. 2:**
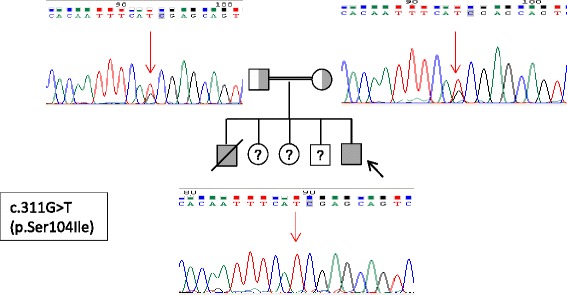
The figure shows segregation of the novel variant by Sanger sequencing in the index case and family members. Footnote: The proband is marked with an arrow. The consanguinity is represented by a double line. Filled and unfilled symbols indicate affected and unaffected individuals, respectively. The parents are represented as a carrier status

We identified a homozygous missense variant (c.311G > T; p.Ser104Ile) in exon 3 of *SLC39A14* (NM_001128431), which was confirmed by Sanger sequencing in both the patient and his affected brother. Both parents were heterozygous carriers of this variant (Fig. 2). Unfortunately, DNA samples from three unaffected siblings were not available for the analysis. This novel variant was not found in HGMD, dbSNP, 1000 Genome project, ExAC database or CIBERER Spanish Variant Server. This variant affected a highly conserved amino acid residue, located in the N-terminus extracellular loop, studied by UCSC browser and Clustal Omega software and it was categorized as pathogenic by SIFT (0.002), PROVEAN (−3.03) and Mutation Taster (142), and as possibly damaging by PolyPhen-2 (0.664).

### Treatment

**Fig. 3:**
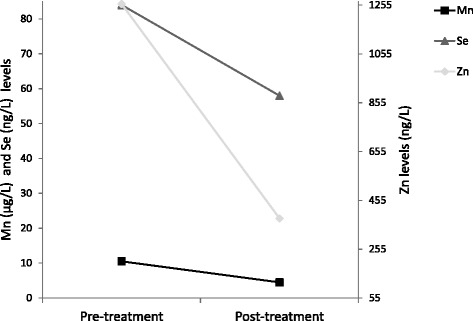
Biochemical analysis before and after treatment. Footnote: Graph shows a reduction of plasma Mn, Zn and Se five days after Na_2_CaEDTA therapy

Following genetic confirmation, we instigated compassionate treatment with the Mn chelator, Na_2_CaEDTA, 1 g/m2/day in two divided oral doses for a five-day course. This protocol was initially proposed for lead intoxication and has more recently been used in patients with *SLC30A10* and *SLC39A14* deficiency [14, 15]. No clinical side effects were recorded during the administration. On the fifth day, we observed a reduction of plasma Mn (from 10.5 to 4.5 μg/L, 57% reduction, RV: 0.4–0.9 μg/L), Zn (from 1260 to 381 ng/L, 69.8% reduction, RV: 628–1200 ng/L) and Se (from 84 to 58 ng/L, 31.5% reduction, RV: 67–104 ng/L) (Fig. 3), and therefore he was supplemented with Zn acetate (10 mg/day) and Se (50 mg every 2 days). There was also a mild decrease in plasma Cu and Fe but values remained within the normal range. Two weeks after treatment, the family referred worsening of cervical dystonia, irritability and sleep difficulties, which improved with incremental doses of diazepam. The family decided to discontinue chelation therapy. Neurological examination 3 weeks later was comparable to baseline.

## Discussion

We present two siblings with a homozygous missense variant in *SLC39A14*, manifesting a rapidly progressive generalized dystonia in infancy and hypermanganesemia, similar to a cohort of eight *SLC39A14* mutated children reported [15]. Our patient did not show polycythemia or liver disease, as observed in SLC30A10-deficient patients (Table 1). We measured plasma and CSF Mn, both showing very high values comparing to our control patients and literature reference ranges [29]. Importantly, Mn values were 3-fold higher in CSF than in plasma; in keeping with the finding that brain Mn levels are four to 20-fold higher in *slc39a14* mutant zebra fish [15] and knockout mice [4, 6] compared to wild-type. Our findings support the hypothesis that brain is likely to be the main organ of Mn deposition in SLC39A14 deficiency.

Previously, Chang et al., 2009 reported a correlation between pallidal index (PI) and plasma Mn concentrations of 43 manganese-exposed welders and concluded that high PI might be attributed to Mn brain deposition [30]. We used PI to quantify pallidal Mn accumulation in our patient, and observed significantly higher values than in controls. Furthermore, our patient had higher PI values compared to occupationally Mn exposed workers [31]. These values slightly increased over time, suggesting the relentless pallidal accumulation of Mn in SLC39A14 deficiency.

Na_2_CaEDTA is a chelating agent that combines with metal ions to form stable and soluble complexes that are excreted in the urine. It ameliorated dystonia and parkinsonism in *SLC30A10* mutated patients [18, 22], and its efficacy proved to be persistent over time is some cases with long-term follow-up [22, 32]. More recently, two *SLC39A14* patients received Na_2_CaEDTA, one, given chelation early in the disease course showed clinical improvement, whereas the other (older) patient continued to deteriorate despite treatment [15]. Interestingly, two *SLC39A14* KO mice models recently demonstrated a positive effect with two different chelators: a zinc-supplemented diet significantly decreased brain Mn uptake [4], and the metal chelator Na2CaEDTA reduced serum Mn levels and rescued motor deficits [10].

The administration of Na_2_CaEDTA in our patient led to the reduction of not only plasma Mn values, but also Se and Zn, which are cofactors of important enzymes such as SOD and selenoproteins, and hence, they were supplemented in our patient. Even though Se is not a cation, the decrease might be an effect of EDTA on renal tubules during chelation therapy [33]. Unfortunately, worsening of dystonia resulted in discontinuation of Na_2_CaEDTA and the long-term efficacy of this treatment could not be tested in this patient.

## Conclusions

In this study we present two infants presenting with dystonia and hypermanganesemia caused by a homozygous missense variant in *SLC39A14*, a recently recognized gene involved in Mn homeostasis in humans, thus expanding the very few descriptions of this disorder. We also report for the first time the elevation of Mn in CSF of *SLC39A14* mutated patients, supporting the hypothesis that brain is an important organ of Mn deposition in SLC39A14-related disease. The measurement of PI values on MRI is a non-invasive method that may help monitor Mn pallidal deposition over time. Finally, we propose that patients with inherited defects of manganese transport should be initially treated with low doses of Na_2_CaEDTA followed by gradual dose escalation, together with a close monitoring of blood trace elements in order to avoid side effects.

## Abbreviations

CDG: Congenital disorder of glycosylation
CSF: Cerebrospinal fluid
Cu: Copper
Mn: Manganese
MRI: Magnetic resonance imaging
PI: Pallidal index
RV: Reference values
Se: Selenium
SIFW: Subcortical frontal white matter
SIGP: Signal intensity in the globus pallidus
SOD: Superoxide dismutase
Zn: Zinc
